# From Hypoxia to Bone: Reprogramming the Prostate Cancer Metastatic Cascade

**DOI:** 10.3390/ijms26157452

**Published:** 2025-08-01

**Authors:** Melissa Santos, Sarah Koushyar, Dafydd Alwyn Dart, Pinar Uysal-Onganer

**Affiliations:** 1 Cancer Mechanisms and Biomarkers Research Group, School of Life Sciences, University of Westminster, London W1W 6UW, UK; m.santos@westminster.ac.uk; 2 UCL Cancer Institute, University College London, Paul O’Gorman Building, 72 Huntley Street, London WC1E 6DD, UK; s.koushyar@ucl.ac.uk (S.K.); a.dart@ucl.ac.uk (D.A.D.)

**Keywords:** prostate cancer, hypoxia, bone metastasis, Wnt signalling, extracellular vesicles, EMT, HIF-1α, therapy resistance

## Abstract

Bone is the most frequent site of distant metastasis in advanced prostate cancer (PCa), contributing substantially to patient morbidity and mortality. Hypoxia, a defining feature of the solid tumour microenvironment, plays a pivotal role in driving bone-tropic progression by promoting epithelial-to-mesenchymal transition (EMT), cancer stemness, extracellular matrix (ECM) remodelling, and activation of key signalling pathways such as Wingless/Integrated (Wnt) Wnt/β-catenin and PI3K/Akt. Hypoxia also enhances the secretion of extracellular vesicles (EVs), enriched with pro-metastatic cargos, and upregulates bone-homing molecules including CXCR4, integrins, and PIM kinases, fostering pre-metastatic niche formation and skeletal colonisation. In this review, we analysed current evidence on how hypoxia orchestrates PCa dissemination to bone, focusing on the molecular crosstalk between HIF signalling, Wnt activation, EV-mediated communication, and cellular plasticity. We further explore therapeutic strategies targeting hypoxia-related pathways, such as HIF inhibitors, hypoxia-activated prodrugs, and Wnt antagonists, with an emphasis on overcoming therapy resistance in castration-resistant PCa (CRPC). By examining the mechanistic underpinnings of hypoxia-driven bone metastasis, we highlight promising translational avenues for improving patient outcomes in advanced PCa.

## 1. Introduction

Prostate cancer (PCa) is the second most diagnosed malignancy among men and a leading contributor to cancer-related mortality worldwide [1,2]. While early-stage, organ-confined PCa can often be effectively managed with surgery or radiotherapy, progression to metastatic and castration-resistant PCa (CRPC) presents a major clinical hurdle [3,4]. Bone is the predominant site of distant metastasis in advanced PCa, occurring in over 80% of affected individuals and contributing to significant skeletal morbidity, treatment resistance, and decreased quality of life [5,6]. Tumour hypoxia, a hallmark of solid cancers, has emerged as a key driver of aggressive disease phenotypes and metastatic spread in PCa, although the extent of this effect can vary depending on tumour subtype, oxygen gradient, and model system used. [7,8]. Resulting from inadequate tumour perfusion and rapid cellular proliferation, hypoxic conditions activate hypoxia-inducible factors (HIFs), particularly HIF-1α, which coordinates the transcription of genes involved in angiogenesis, metabolic reprogramming, invasion, and immune modulation [9,10]. These adaptations support tumour cell survival under stress and enable progression to a more malignant state.

Hypoxia modulates multiple oncogenic pathways that promote bone-tropic dissemination in preclinical PCa models, although the relevance and consistency of these pathways in clinical bone metastasis remains an area of active investigation. In this context, hypoxia has an influence on epithelial-to-mesenchymal transition (EMT), cancer stem cell (CSC) maintenance, extracellular matrix (ECM) remodelling, and activation of Wnt/β-catenin, Notch, and PI3K/Akt signalling cascades [11,12,13]. EMT allows epithelial PCa cells to acquire mesenchymal traits, including enhanced motility and invasiveness, which are essential for intravasation into the circulation and subsequent extravasation at metastatic sites [14]. In PCa, hypoxic regions within the tumour microenvironment (TME) maintain a population of PCa stem cells (PCSCs), characterised by enhanced self-renewal, resistance to androgen deprivation therapy (ADT) and radiotherapy, and these PCSCs are increasingly recognised as key mediators of metastatic competence (particularly to bone) and tumour recurrence. [15,16]. These cellular and molecular transformations are further supported by dynamic changes in the TME, including altered ECM composition and increased stromal remodelling [15].

Moreover, hypoxic stress enhances the biogenesis and secretion of extracellular vesicles (EVs), that transport regulatory proteins, lipids, mRNAs and non-coding RNAs to both local and distant cellular targets, shaping the pre-metastatic niche and influencing metastatic organotropism [17,18,19]. EVs derived from hypoxic PCa cells have been shown to carry pro-metastatic cargo, including matrix metalloproteinases (MMPs), integrins, and microRNAs (miRs/miRNAs) that modulate gene expression in recipient cells, promoting a supportive microenvironment for bone colonisation. Furthermore, hypoxia-induced expression of bone-homing molecules such as CXCR4, integrin αvβ3, and PIM kinases enhances tumour cell adhesion to bone matrix components and eases extravasation into the bone marrow niche, where they interact with osteoblasts and osteoclasts to establish osteoblastic or mixed lesions [20,21,22].

Despite growing insights into the role of hypoxia in PCa, the mechanisms linking hypoxic signalling to bone-specific metastasis remain incompletely understood. This review synthesises current evidence on how hypoxia promotes metastatic progression in PCa, with particular attention to bone tropism. We examine the crosstalk between HIF signalling and downstream pathways, including Wnt/β-catenin, Notch, and PI3K/Akt, that regulate EMT, cancer stemness, and EV biogenesis.

We further highlight the role of hypoxia-induced bone-homing molecules such as CXCR4, integrins, and PIM kinases in facilitating skeletal colonisation. Finally, we explore emerging therapeutic strategies targeting these pathways, aiming to overcome treatment resistance and improve outcomes for patients with advanced PCa.

## 2. Hypoxia-Mediated Mechanisms Driving Bone-Tropic PCa

**Figure 1:**
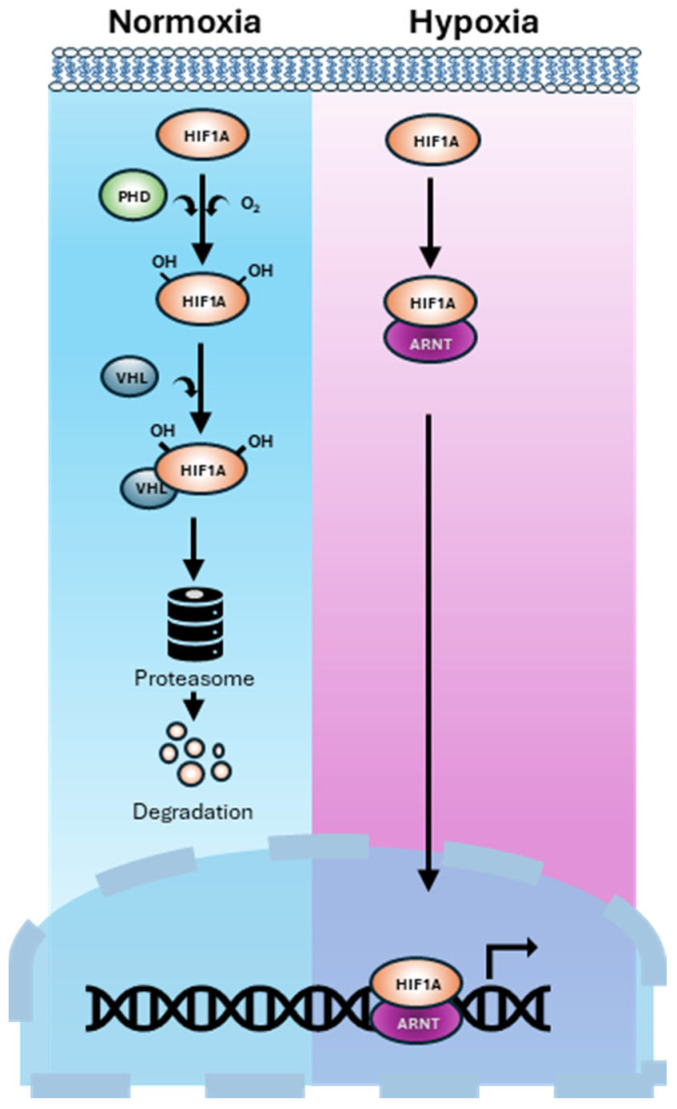
HIF the master regulator of the cellular response to low oxygen (hypoxia). Under normoxia, HIF-1α is hydroxylated by PHD enzymes. Hydroxylated HIF-1α binds to VHL protein. This complex is ubiquitinated and degraded by the proteasome. Under Hypoxia, PHD enzymes are inactive due to a lack of oxygen. HIF-1α escapes degradation, accumulates, and translocates to the nucleus where it dimerises with HIF-β (known as ARNT) and binds to hypoxia response elements in DNA. This activates transcription of hypoxia adaptive pathways.

HIF-1α is a master transcriptional regulator that enables cellular adaptation to oxygen deprivation [23]. Under normal oxygen levels, HIF-1α is hydroxylated by prolyl hydroxylase domain (PHD) enzymes and targeted for proteasomal degradation via von Hippel–Lindau (VHL) protein [24]. In hypoxia, this degradation is inhibited, allowing HIF-1α to accumulate, dimerise with HIF-1β (ARNT), and activate a broad array of genes involved in angiogenesis, metabolism, and survival [25,26] (Figure 1). This stabilisation of HIF-1α initiates diverse downstream signalling events that drive tumour progression and metastasis in PCa [27,28].

**Figure 2:**
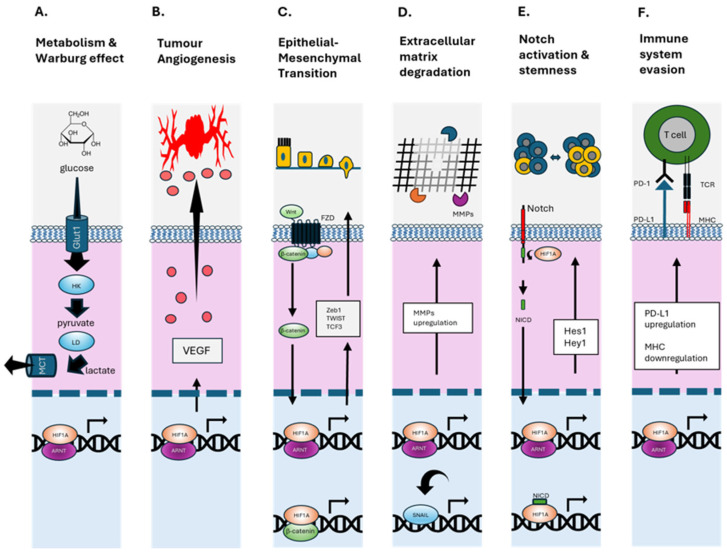
HIF-1α regulated cellular functions. (**A**) Upregulates glucose importer (GLUT1), increases glycolytic flux (e.g., hexokinase HK & lactate dehydrogenase LD) and conversion to lactate (exported via MCT1), supporting rapid cell growth and survival in hypoxic environments (Warburg Effect). (**B**) Binds to hypoxia response elements in the VEGF promoter, enhancing its expression and stimulating angiogenesis. (**C**) Promotes EMT by activating the transcription factors—Snail, Slug, TWIST, and ZEB1/2—reducing cell–cell adhesion and polarity, and increasing motility and invasiveness. Enhances Wnt/β-catenin signalling by promoting β-catenin nuclear localisation and transcriptional activity. (**D**) Enhances the expression of MMPs that degrade the extracellular matrix. These changes collectively facilitate cancer cell migration, invasion, and metastasis under hypoxic conditions. (**E**) Enhances Notch receptor expression and activation of the Notch intracellular domain (NICD), which translocates to the nucleus to influence transcription of genes in angiogenesis, stem cell maintenance, and EMT. (**F**) Upregulates PD-L1 expression on tumour, which binds to PD-1 on T cells, suppressing their cytotoxic activity and promoting T cell exhaustion. Negatively regulates MHC class I expression, contributing to immune evasion in hypoxic tumour environments by less T cell receptor (TCR) binding. Up and down arrows indicate general movement of proteins in the cytoplasm and nucleus.

Hypoxia drives a cascade of biological processes that converge to promote PCa metastasis, particularly to the bone (Figure 2). At the molecular level, HIF-1α plays a central role in orchestrating transcriptional responses to low oxygen tension [29]. Upon stabilisation, HIF-1α induces a suite of genes involved in angiogenesis (Figure 2B, VEGF, ANGPT2, SDF1, and SCF [11]), glucose metabolism (Figure 2A, GLUT1), and invasion (Figure 2D, MMPs), all of which are crucial to tumour progression and metastatic competence [30,31]. Under hypoxic conditions, HIF-1α directly induces MMP9 transcription, facilitating basement membrane degradation and tumour cell extravasation (Figure 2D) [32,33]. In PCa, HIF-1α overexpression is associated with enhanced invadopodia formation, EMT (Figure 2C), and increased MMP9 activity, which correlates with bone metastatic potential [34]. Additionally, MMP9 downregulates COL4A1, further promoting ECM degradation and tumour dissemination [35]. In the hypoxic bone marrow, MMP9 is involved not only in ECM breakdown but also in osteoclast activation, supporting osteolytic activity and tumour growth [35,36]. MMP7 contributes by solubilising RANKL, promoting osteoclastogenesis and bone degradation [37,38]. The interplay between MMPs and bone-resorptive mechanisms forms a positive feedback loop that accelerates metastatic colonisation [39].

Hypoxia-induced EVs from PCa cells have been shown to carry active MMP2 and MMP9, which remodel the ECM at pre-metastatic niches, preparing distant bone environments for tumour seeding (Figure 2D). These vesicles also support angiogenesis and immune evasion (Figure 2F), further enhancing metastatic competency [40].

One of the hallmark features of hypoxia is its ability to induce EMTs (Figure 2C). HIF-1α promotes EMT in part by upregulating signalling cascades such as Wnt/β-catenin and Notch, which subsequently induce the expression of key EMT-associated transcription factors including Snail, Twist and ZEB1 [41]. These changes increase the ability of PCa cells to disseminate from the primary tumour and invade distant tissues, including bone [42]. In parallel, hypoxia enriches the subpopulation of CSCs, characterised by markers such as CD44, ALDH1, and OCT4 (Figure 2E). These cells exhibit self-renewal, pluripotency, and resistance to standard therapies, contributing to tumour recurrence and metastasis [9]. Hypoxia not only maintains the CSC phenotype via HIF-dependent mechanisms but also increases cellular plasticity, allowing non-stem cells to acquire stem-like features under low oxygen tension [9,43]. This plasticity enhances tumour heterogeneity and adaptability, further supporting metastatic seeding [44]. EVs represent another critical conduit through which hypoxia enhances metastatic potential. Hypoxic PCa cells release EVs enriched with oncogenic cargo, such as miR-210, miR-21, HIF1α target proteins, and long non-coding RNAs, that modulate the behaviour of recipient cells in the tumour microenvironment and distant pre-metastatic niches [45] (Figure 2F). These EVs have been shown to activate Wnt/β-catenin signalling, promote EMT (Figure 2C), suppress immune responses (Figure 2F), and facilitate stromal reprogramming in bone, making them key mediators of organotropism [45,46].

Similar findings have been observed in our ongoing work on hypoxic PCa-derived EVs and their role in modulating Wnt signalling and EMT in prostate epithelial cells. Together, these hypoxia-induced processes, EMT, CSC enrichment, and EV-mediated signalling, form a coordinated axis that enables PCa cells to escape the primary tumour, survive in circulation, and adapt to the bone microenvironment [47,48]. This complex interplay between intracellular reprogramming and extracellular communication underpins the aggressive metastatic behaviour observed in advanced PCa [49].

## 3. Bone-Homing Molecules and Hypoxic Modulation

Hypoxia enhances the expression and activity of bone-homing molecules that facilitate the preferential localisation of PCa cells to the bone microenvironment [43]. Among these, the CXCR4/CXCL12 axis is particularly important [42,50]. In PCa, hypoxia induces CXCR4 expression via HIF-1α–dependent transcription, enhancing tumour cell chemotaxis toward CXCL12 (SDF-1) secreted by bone marrow stromal cells, a key mechanism promoting bone metastasis [51]. This chemokine-guided migration supports directed invasion and anchoring within the bone niche. Integrins, particularly αvβ3 and α6β1, are overexpressed in PCa and have been associated with tumour growth, angiogenesis, and metastasis. These integrins contribute to bone metastasis by mediating prostate cancer cell adhesion to extracellular matrix components such as fibronectin and osteopontin, which are abundant in the bone microenvironment [52,53]. Hypoxia-induced integrin expression enhances tumour cell survival, proliferation, and resistance to apoptosis under anchorage-independent conditions [51]. These integrin-mediated interactions not only support metastatic colonisation but also initiate signalling pathways that promote osteomimicry, a process by which tumour cells adopt bone-like phenotypes to evade immune surveillance and adapt to the osseous microenvironment [54]. Another family of hypoxia-responsive proteins implicated in bone metastasis are PIM kinases, particularly PIM1 and PIM2 [55]. These serine/threonine kinases promote tumour growth, survival, and metabolic adaptation in hypoxic conditions [56]. Elevated expression of PIM kinases in PCa has been associated with enhanced metastatic potential and poor prognosis [57,58]. They act downstream of both HIF-1α [59] and STAT3, bridging hypoxic stress responses with oncogenic signalling [58]. Collectively, the hypoxia-mediated regulation of CXCR4, integrins, and PIM kinases enables PCa cells to efficiently traffic to, colonise, and persist within the bone microenvironment [20,55]. Targeting these molecules offers potential therapeutic avenues for limiting skeletal metastases and improving disease outcomes.

Importantly, hypoxia appears to play distinct roles at different stages of the metastatic cascade, from the initial ‘seeding’ of tumour cells in bone to their later ‘colonisation’ and expansion [20]. During the early seeding phase, hypoxia-induced upregulation of CXCR4 and integrins facilitates chemotaxis, adhesion, and survival in the hostile, low-oxygen bone niche [60]. This allows circulating tumour cells to anchor effectively and resist anoikis [61].

At later stages, once tumour cells have seeded in the bone marrow, hypoxia continues to support metastatic outgrowth by sustaining PIM kinase signalling, enhancing integrin-mediated osteomimicry, and maintaining metabolic flexibility under nutrient and oxygen-deprived conditions [62]. These adaptations allow PCa cells not only to persist but also to remodel their microenvironment, suppress immune clearance, and engage in reciprocal signalling with osteoblasts and osteoclasts [42,63].

Hypoxia acts as both an enabler of bone homing and a sustainer of metastatic colonisation, informing therapeutic timing and drug target selection. Intervening early to block seeding versus disrupting late-stage colonisation may require different strategies, even if the underlying hypoxic pathways overlap [64].

## 4. Therapeutic Targeting of Hypoxia-Driven Bone Metastasis

**Figure 3:**
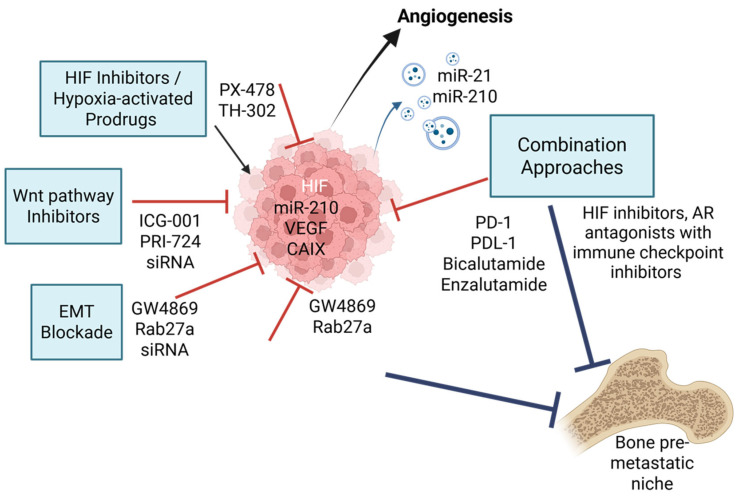
Targeted therapeutic strategies against hypoxia-induced EV signalling and bone metastasis. Hypoxia in the PCa tumour microenvironment stabilises HIF-1α, which transcriptionally upregulates pro-metastatic factors such as VEGF (angiogenesis), miR-210 (cell survival), and CAIX (pH regulation). This drives EMT, immune evasion, and release of EVs loaded with oncogenic and immunomodulatory cargo. Targeted therapies include HIF-1α inhibitors, (PX-478), hypoxia-activated prodrugs (TH-302), Wnt/β-catenin and EMT blockers (PRI-724, ICG-001, ZEB1/Snail siRNAs), and EV biogenesis inhibitors (GW4869, Rab27a blockade). The diagram illustrates how these processes promote pre-metastatic niche formation and bone colonisation. Combination approaches integrating hypoxia-targeted agents with immune checkpoint inhibitors or AR antagonists offer promising avenues for combating bone metastasis in CRPC. Red T-bar arrows indicate direct molecular inhibition of hypoxia-induced pathways and blue T-bar arrows indicate inhibition of downstream metastatic processes. Created by BioRender Science Suite Inc (Toronto, ON, Canada).

The hypoxic TME presents both a challenge and an opportunity for therapeutic intervention in advanced PCa (Figure 3). By targeting the molecular drivers and downstream consequences of hypoxia, several strategies aim to prevent or delay bone metastasis and improve outcomes in CRPC.

### 4.1. HIF Inhibitors and Hypoxia-Activated Prodrugs

Given the central role of HIF-1α in orchestrating hypoxia-adaptive responses, small-molecule HIF inhibitors have been explored as potential therapies. Agents such as PX-478 have shown efficacy in preclinical PCa models by inhibiting HIF-1α expression, reducing angiogenesis, and impairing tumour growth [65,66,67]. However, clinical translation has been limited by off-target effects and modest efficacy as monotherapy [68]. An alternative approach involves hypoxia-activated prodrugs (HAPs), such as TH-302 (evofosfamide), which remain inert under normoxia but become cytotoxic in hypoxic regions. These agents selectively target hypoxic tumour zones, reducing systemic toxicity while enhancing antitumour efficacy [69,70].

Emerging HIF inhibitors such as PT2385 (targeting HIF-2α) and the dual HIF-1α/p300 disruptors have shown enhanced specificity and tumour selectivity [71]. Recent efforts focus on combining HIF inhibitors with immune checkpoint inhibitors or anti-angiogenic agents to synergistically overcome resistance. Additionally, imaging modalities that detect tumour hypoxia in vivo, such as [18F]-fluoromisonidazole (FMISO) PET, may guide patient stratification and improve therapeutic precision [72].

### 4.2. EMT and Wnt Pathway Inhibitors

As EMT is a key mechanism driving metastasis under hypoxia, therapeutic strategies aimed at reversing or inhibiting EMT have gained interest [49]. Pharmacologic agents targeting TGF-β, Notch, and Wnt/β-catenin signalling pathways are under investigation in several cancer types. Wnt inhibitors, such as LGK974 and PRI-724, have shown preclinical efficacy in suppressing EMT and stemness in colorectal, breast, and pancreatic cancer models. Early-stage studies are beginning to explore their relevance in PCa, particularly in castration-resistant contexts [73,74]. In PCa models, these inhibitors have demonstrated the ability to impair bone colonisation and sensitise tumours to ADT [73,74].

More recently, combination approaches targeting both EMT and Wnt signalling have been investigated to counteract plasticity-driven resistance. For example, ICG-001, which blocks the β-catenin/CBP interaction, has been shown to reverse castration resistance and restore AR signalling control [75,76]. Targeting transcription factors like ZEB1 or Snail using siRNA-loaded nanoparticles also holds promise for halting EMT progression [77]. These strategies may be particularly effective in halting early dissemination and enhancing response to systemic therapies.

### 4.3. Extracellular Vesicle-Based Therapeutics

Targeting EV release or uptake represents a novel strategy to disrupt tumour communication under hypoxic conditions [78,79]. Agents such as GW4869 (a neutral sphingomyelinase inhibitor) can block EV biogenesis, reducing the transfer of oncogenic cargo between tumour and stromal cells [80,81]. Additionally, engineered EVs are being explored as delivery vehicles for siRNAs, immune stimulants, or small molecule inhibitors to reprogram the tumour microenvironment or enhance antitumour immunity [82].

Efforts to selectively disrupt hypoxia-induced EV release have identified Rab27a, nSMase2, and HIF-1α–regulated exosome biogenesis pathways as viable targets [83]. In vivo studies have shown that systemic blockade of EV trafficking can reduce pre-metastatic niche formation in bone and limit tumour-derived immunosuppression [83]. Meanwhile, engineered exosomes delivering CRISPR/Cas9 or miRNA antagonists targeting oncogenic EV cargo (e.g., miR-210, miR-21) are being developed as next-generation precision tools for metastatic PCa [84,85].

### 4.4. Combination Strategies and Future Directions

Given the multifaceted role of hypoxia in PCa progression, combination therapies targeting multiple pathways simultaneously are required for durable clinical benefit. Monotherapies targeting isolated pathways have shown limited efficacy in advanced disease settings [86]. For example, co-targeting HIF signalling and Wnt/β-catenin has demonstrated promising results in preclinical models using PCa cell lines, disrupting the cooperative effect of these pathways on EMT and cancer stemness [87]. In parallel, combining hypoxia-activated prodrugs such as evofosfamide or tarloxotinib with immune checkpoint inhibitors may enhance tumour immunogenicity by alleviating hypoxia-induced immunosuppression and restoring T-cell function [88].

Specifically designed clinical trials that incorporate validated biomarkers of tumour hypoxia are critical to optimising these therapeutic combinations. Biomarkers such as CAIX, GLUT1, and miR-210 have demonstrated utility in stratifying PCa patients and predicting therapeutic responses [89,90]. For instance, CAIX overexpression has been associated with biochemical recurrence and poor prognosis in PCa, supporting its role as both a prognostic and predictive marker [87,91,92]. Similarly, elevated GLUT1 expression is strongly correlated with tumour hypoxia, glycolytic metabolism, and resistance to conventional therapies, making it an attractive candidate for patient selection and monitoring [91]. miR-210, a key hypoxia-regulated microRNA, is detectable in circulation and has emerged as a robust non-invasive biomarker with diagnostic and prognostic potential, especially when used in liquid biopsy platforms [93].

A growing number of preclinical and clinical studies are now focused on integrating these hypoxia-targeted agents into broader therapeutic regimens. Table 1 provides an overview of emerging therapeutic agents and strategies aimed at disrupting hypoxia-driven tumour progression and bone metastasis in PCa. Future research should prioritise adaptive trial designs, biomarker-led patient selection, and longitudinal monitoring of hypoxia dynamics to maximise therapeutic impact.

### 4.5. Hypoxia and Immunotherapy Resistance in PCa

Immunotherapy is changing the treatment landscape of cancer therapy, with great advances in the treatment of leukaemia and lymphoma [100]. Immunotherapy is also beginning to show some clinical benefit in solid cancers such as melanoma and renal cancers [101,102]; however, it has yet to make a significant impact on solid tumours such as PCa.

PCa is characterised as being “immunologically cold”, with low levels of tumour-infiltrating lymphocytes, high levels of immunosuppressive cells, and low neoantigen expression [103]. Prostate tumours express immune checkpoint molecules such as PD-L1, show presence of T-cell exhaustion, and accumulation of immunosuppressive cell populations (Tregs), all of which contribute to diminished immune responses [104].

Although not regarded as being the most hypoxic tumour, PCa hypoxia levels increase with clinical stage and patient age [105]. Even the hyperproliferation of prostate epithelial cells, in situ, driven by loss of the tumour suppressor, PTen, is sufficient for the activation and accumulation of HIF-1α at the very early prostate intraepithelial neoplasia (PIN) stage, with inflammatory and HIF-1α-driven miRNA expression [106,107].

CXCR4, through its interaction with its ligand CXCL12, contributes significantly to immunosuppression within the TME [108]. This signalling axis plays a dual role: it promotes tumour progression and simultaneously shapes an immunosuppressive niche that hinders effective anti-tumour immune response [108,109]. One key mechanism involves the recruitment and retention of immunosuppressive cell types, such as Tregs, myeloid-derived suppressor cells (MDSCs), and CXCR4hi neutrophils. These cells suppress cytotoxic T lymphocyte (CTL) activity and dampen the immune system’s ability to recognise and destroy tumour cells [110,111]. HIF1a upregulates CD47 expression, allowing tumour cells to avoid phagocytosis by macrophages and contributes to resistance against immunotherapy [112,113].

Additionally, the PD-L1 receptors (programmed cell death 1 ligand 1) which inhibits T-cell activation and proliferation, is specifically upregulated by HIF-1α binding to its promoter [110].

PD-L1 binds to PD-1 on T cells, suppressing their activity and enabling tumour survival. This interaction contributes to an immunosuppressive microenvironment, reducing cytotoxic T cell function and recruiting regulatory T cells. Studies show that disrupting hypoxic zones sensitises prostate tumours to PD-1 blockade therapies, enhancing immune response [114]. Thus, targeting both hypoxia and PD-1 pathways may improve outcomes in prostate cancer by reversing immune suppression and boosting immunotherapy efficacy.

Hypoxia itself also downregulates the antigen-presenting MHC class 1 molecules on cancer cells which may allow tumour cells to escape from immune detection [112]. HIF-1α suppresses transcription of MHC class I heavy chains and antigen-processing components like TAP1/2 and LMP7, reducing surface presentation of tumour antigens [112], leading to diminished CD8+ T cell infiltration and impaired cytotoxic responses.

ADT has been shown to enhance the immunogenicity of PCa [115]; however, this is often very transient, with the rapid development of drug resistance and immune evasion mechanisms. PCa often fail to generate immune responses, even at high tumour burden, and hence the poor response to immunotherapies presents a major challenge with strategies requiring methods to induce an immune hot PCa tumour and its microenvironment.

## 5. Knowledge Gaps and Research Priorities

Although considerable progress has been made in understanding the role of hypoxia in driving PCa progression and bone metastasis, several critical knowledge gaps persist. Current understanding is primarily derived from preclinical models, and the context-dependence of hypoxic signalling, such as the timing, severity, and duration of hypoxia, has not been defined [43,116,117]. In particular, the precise in vivo mechanisms by which EVs, PIM kinases, and bone-homing molecules mediate metastatic colonisation require further investigation in clinically relevant systems [118]. Another major limitation is the lack of validated, non-invasive biomarkers for tumour hypoxia in PCa. This impedes efforts to stratify patients who may benefit from hypoxia-targeted therapies or participate in hypoxia-guided clinical trials [119,120]. Additionally, while hypoxia contributes to immune evasion, few studies have evaluated how its immunosuppressive effects can be reversed through combination approaches involving immune checkpoint blockade or other immunomodulatory interventions [121,122]. To address these issues, integrative research efforts are needed that incorporate advanced model systems, real-time hypoxia imaging, and systems-level analyses of TME interactions. Table 2 summarises key areas of uncertainty and outlines future directions that may improve our mechanistic understanding and therapeutic targeting of hypoxia in bone-metastatic PCa.

## 6. Conclusions and Future Perspectives

Bone metastasis remains a major cause of morbidity in advanced PCa [130], and hypoxia is now recognised as a key orchestrator of the molecular events that drive this process. Through the activation of HIF-1α and downstream pathways, hypoxia induces EMT, enhances cancer stem cell plasticity, remodels the tumour microenvironment, and promotes the secretion of pro-metastatic EVs [43,131]. These changes work in concert with upregulation of bone-homing molecules such as CXCR4, integrins, and PIM kinases to facilitate skeletal colonisation [55,132]. Notably, hypoxia-driven extracellular vesicles not only mediate local invasion but also influence distant stromal environments, preconditioning the bone niche for successful tumour engraftment.

An improved understanding of hypoxia-driven mechanisms offers several therapeutic opportunities. Targeting HIF signalling, inhibiting EMT and Wnt pathways, disrupting EV-mediated communication, and employing hypoxia-activated prodrugs represent promising strategies to counteract metastasis and overcome treatment resistance [133]. Moreover, the development of hypoxia-responsive drug delivery systems and nanocarriers could enable more precise targeting of hypoxic tumour zones while minimising off-target effects [134].

Future research should prioritise integrative, biomarker-driven approaches that address tumour heterogeneity and the complex interplay between cancer cells and their microenvironment. This includes the incorporation of non-invasive hypoxia markers such as circulating miRNAs or imaging-based surrogates to monitor treatment response in real-time. Ultimately, the translation of hypoxia-targeted therapies into clinical benefit will require multidisciplinary collaboration, robust preclinical models that mimic the hypoxic bone metastatic niche, and well-designed clinical trials with rational patient stratification. By leveraging insights into hypoxic adaptation, we can develop novel, personalised strategies to delay or prevent bone metastases and improve outcomes for patients with metastatic PCa.

## Figures and Tables

**Table 1:** Clinical trials investigating the role of hypoxia in prostate cancer.

Drug	Mechanism of Action	Study Type	Study Details	Clinical Trial ID	Trial Start Date	Study Completion Date	References
PR-104	Hypoxia activated pro-drug	Phase Ib	Non-randomised, open label intervention study assessing the side effects and optimal dose of PR-104 when given in combination with Docetaxel or Gemcitabine in advanced solid cancers. Prostate cancer patients (*n* = 4).	NCT00459836	2007	2009	[94,95]
N/A	N/A	Observational	Prospective study assessing molecular features of tumour hypoxia in combination with morphological and functional MRI data and the presence of micro metastases. Patients are assessed longitudinally for clinical outcomes such as recurrence, metastatic disease and death.	NCT01464216	2011	Estimated 2030	[96]
Pimonidazole	Hypoxia specific marker	Observational	Open label study interventional study investigating hypoxia and stem cell content in prostate cancer. Prostate cancer patients who have agreed to an open radical prostatectomy are enrolled into this study. Primary objective is to quantify Pimonidazole staining in radical prostatectomy specimens as a primary determinant of biochemical failure.	NCT02095249	2014	Estimated 2028	[97]
non-investigational medicinal product (IMP) pimonidazole	Hypoxia specific marker	Observational	Prospective, non-randomised, exploratory biopsy and imaging biomarker study. Primary aim is to determine the association between hypoxia in the primary tumour with the presence of skeletal metastases. Primary objective is to identify differences in genomic aberrations samples with and without hypoxia between hormone naïve prostate cancer and paired skeletal metastases.	NCT05702619	2021	2023	[98]
Evofosfamide (IMGS-101)	Hypoxia activated pro-drug	Phase I/II	Non-randomised, open label intervention study assessing the overall safety, tolerability and effectiveness of the combination of IMGS-101 with Zalifrelimab, and Balstilimab (immunotherapies) in solid cancers, including metastatic castration resistant prostate cancer.	NCT06782555	2025	Estimated 2028	[99]

**Table 2:** Knowledge Gaps and Research Priorities in Hypoxia-Driven Prostate Cancer Bone Metastasis.

Knowledge Gap	Research Priority	References
Lack of validated biomarkers for hypoxia in PCa	Develop non-invasive tools such as circulating hypoxia-associated miRNAs (such as miR-210) or FMISO PET imaging for patient stratification	[123]
Limited in vivo understanding of hypoxia-induced EV cargo and function	Elucidate the organ-specific roles of EV content using lineage-tracing, EV-labelling, and preclinical metastasis models	[124]
Unclear role of PIM kinases in mediating skeletal colonisation	Investigate how hypoxia-regulated PIM1/2 influence bone homing and osteomimicry in PCa cells	[55]
Context-dependent effects of hypoxia (e.g., dose, duration, microenvironment)	Compare acute vs. chronic hypoxia across PCa models, using varying oxygen gradients and tumour stages	[125]
Challenges in targeting the CXCR4/CXCL12 axis therapeutically	Dissect spatial and temporal expression dynamics of CXCR4 under hypoxia to optimise therapeutic targeting	[126]
Poor immunotherapy efficacy in hypoxic PCa	Explore rational combinations of immune checkpoint inhibitors with HIF, VEGF, or CXCR4 inhibitors	[127,128]
Lack of precision delivery systems for hypoxia-targeted agents	Develop tumour-selective nanocarriers or exosome-based platforms responsive to hypoxic stimuli	[118,129]

## Data Availability

No new data were created or analysed in this study. Data sharing is not applicable to this article.

## Abbreviations

The following abbreviations are used in this manuscript:

ADT	Androgen deprivation therapy
Akt	Protein kinase B
ALDH1	Aldehyde dehydrogenase 1
AR	Androgen receptor
CAIX	Carbonic anhydrase IX
CBP	CREB-binding protein
CD44	Cluster of differentiation 44
COL4A1	Collagen type IV alpha 1 chain
CRISPR	Clustered regularly interspaced short palindromic repeats
CRPC	Castration-resistant prostate cancer
CSC	Cancer stem cell
CXCL12	C-X-C motif chemokine ligand 12
CXCR4	C-X-C chemokine receptor type 4
ECM	Extracellular matrix
EMT	Epithelial-to-mesenchymal transition
EV	Extracellular vesicle
FMISO	Fluoromisonidazole
GLUT1	Glucose transporter 1
HAP	Hypoxia-activated prodrug
HIF	Hypoxia-inducible factor
HIF-1α	Hypoxia-inducible factor 1 alpha
HK	Hexokinase
LD	Lactate dehydrogenase
lncRNA	Long non-coding RNA
MCT1	Monocarboxylate transporter 1
MHC	Major histocompatibility complex
miR	MicroRNA
MMP	Matrix metalloproteinase
NICD	Notch intracellular domain
nSMase2	Neutral sphingomyelinase 2
Oct-4	Octamer-binding transcription factor 4
PCa	Prostate Cancer
PD-L1	Programmed death-ligand 1
PET	Positron emission tomography
PI3K	Phosphoinositide 3-kinase
PIM	Proviral integration site for Moloney murine leukemia virus
PIN	Prostatic intraepithelial neoplasia
PTEN	Phosphatase and tensin homolog
RANKL	Receptor activator of nuclear factor kappa-B ligand
siRNA	Small interfering RNA
STAT3	Signal transducer and activator of transcription 3
TCR	T cell receptor
TGF-β	Transforming growth factor beta
Treg	Regulatory T cell
VEGF	Vascular endothelial growth factor
